# Supplementary material for: Lysine/RNA-interactions drive and regulate biomolecular condensation
Source: Nat Commun. 2019 Jul 2;10:2909. doi: 10.1038/s41467-019-10792-y (PMC6606616; doi:10.1038/s41467-019-10792-y)
Supplement: Supplementary file 1 — Supplementary Information [file 41467_2019_10792_MOESM1_ESM.pdf]

Supplementary Information

Lysine/RNA-interactions drive and regulate biomolecular condensation

T. Ukmar-Godec et al.

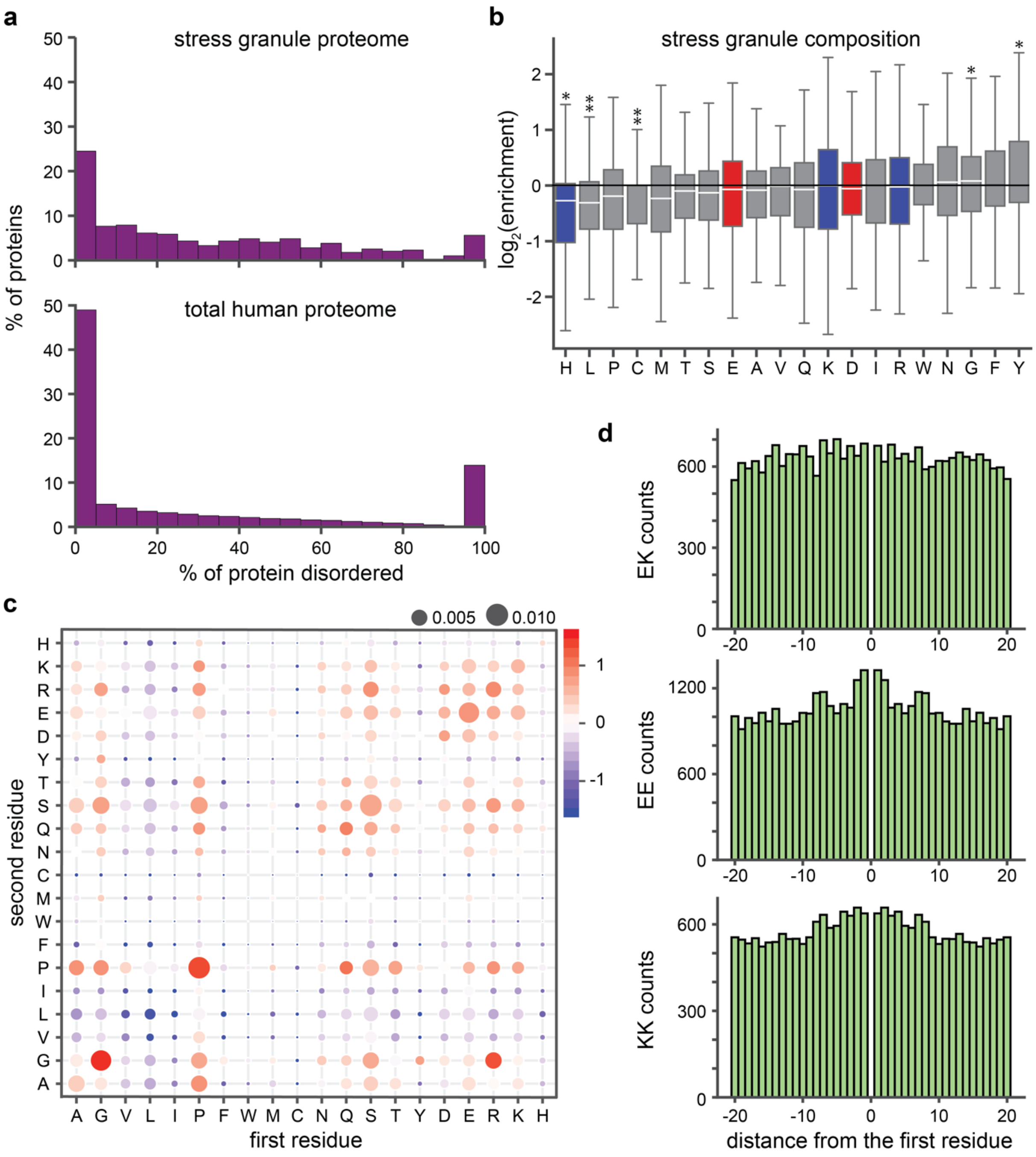

Supplementary Figure 1. Composition of disordered regions of proteins in stress granules. **a** Distribution of the fraction of disorder predicted by IUpred for stress granule proteins (top) and the entire human proteome (bottom). **b** Box plot of log2-fold enrichment of amino acids in disordered regions of stress granules relative to disordered regions of the entire human proteome. Boxes and whiskers show, from bottom to top, 5%, 25%, 50%, 75% and 95% quantiles. Red/blue: positively/negatively charged residues. P-values $<10^{-5}$, *, $<10^{-10}$: **. **c** Log2 enrichment (red) or depletion (blue) of dipeptides in disordered regions of stress granules relative to disordered regions of the entire human proteome. First residue on x-axis, second on y-axis. Size of circles: total probability of dipeptide (sum=1). **d** Same charges cluster together in stress granules, while opposite charges do not. Top: Total counts of amino acid K at position x relative to an E at position 0 (first residue) in disordered regions of stress granules. Middle and bottom: Same for K / K and E / E instead of K / E. Residues of same charge are enriched within +/- 5 residues of each other. Source data are provided as a Source Data file.

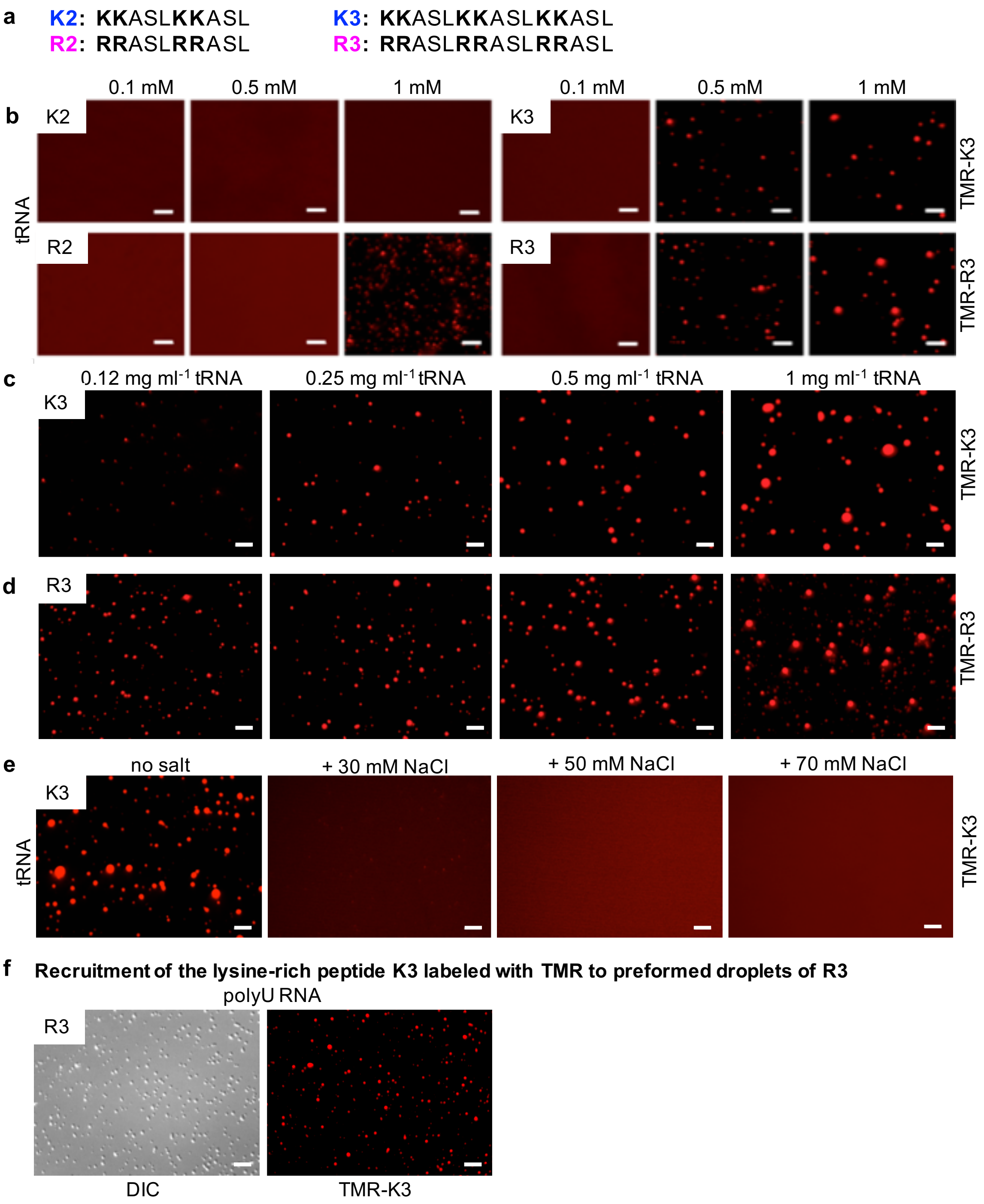

Supplementary Figure 2. Influence of RNA, peptide and salt concentration on coacervation of lysine- and arginine-rich peptides**. a** Amino acid sequences of lysine- (K2, K3) and arginine-rich (R2, R3) peptides. **b** Fluorescence images of K2 and K3 (top), and R2 and R3 (bottom) depicting the influence of peptide concentration (0.1 mM, 0.5 mM, 1 mM) at fixed tRNA concentration (0.5 mg ml^{-1}) on droplet formation. **c, d** Fluorescence images depicting the influence of tRNA concentration on the phase behavior of K3 (1 mM; **c**) and R3 (1 mM; **d**). tRNA concentrations are listed on the top. **e** Ionic strength-dependence of liquid phase separation of the K3 peptide (1 mM K3, 1 mg ml^{-1} tRNA in 50 mM HEPES, pH 7.4). Added NaCl is indicated on the top. **f** The peptide TMR-K3 is recruited into preformed R3/RNA-droplets (0.5 mM R3, 0.5 mg ml^{-1} polyU RNA in 50 mM HEPES, pH 7.4). For fluorescence microscopy, small amounts (see methods section) of TMR-K3 or TMR-R3 were added. Scale bars, 10 µm.

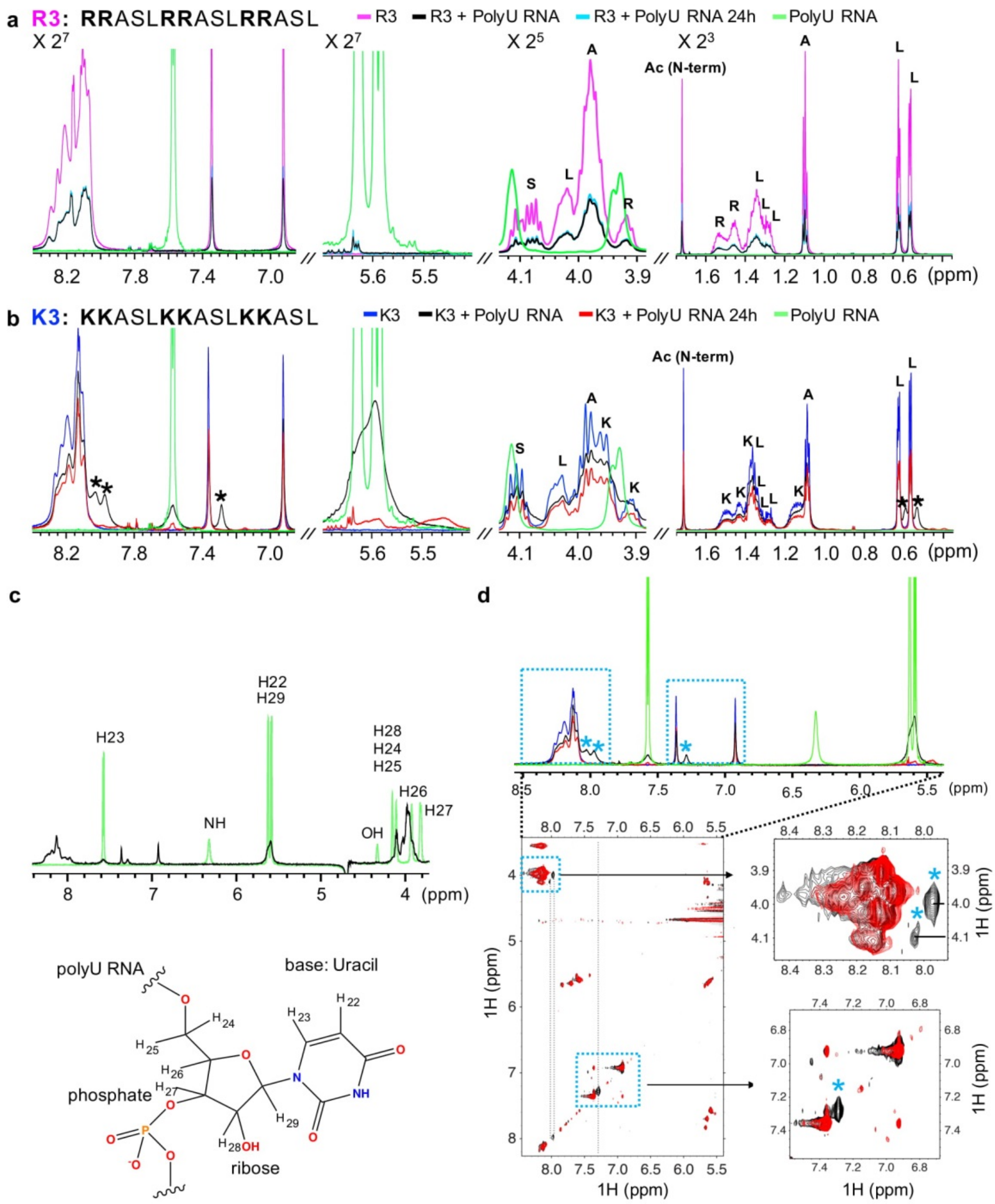

Supplementary Figure 3. Comparison of NMR spectra of R3 and K3 peptides in the monomeric dispersed phase and in the phase separated states. **a** 1D ^{1}H NMR spectra of the peptide sample R3 (1 mM; magenta), R3 with polyU RNA forming liquid droplets (1 mM R3 + 1 mg ml^{-1} polyU RNA; black), the same sample after 24 h (light blue), and the control sample of polyU RNA (1 mg ml^{-1}; green). **b** 1D ^{1}H NMR spectra of the peptide K3 (1 mM; blue), K3 with polyU RNA forming liquid droplets (1 mM K3 + 1 mg ml^{-1} polyU RNA; black) and the same sample after 24 h (red), as well as polyU RNA alone (1 mg ml^{-1}; green). **c** (top) 1D NMR spectra of polyU RNA (1 mg ml^{-1}; green) alone and polyU RNA with K3 (1 mg ml^{-1} polyU RNA + 1 mM K3; black). Assignments of RNA resonances are indicated. (bottom) Chemical structure of polyU RNA. **d** (top) 1D ^{1}H NMR spectrum of the peptide K3 (1 mM; blue), K3 with polyU RNA forming liquid droplets (1 mM K3 + 1 mg ml^{-1} polyU RNA; black) and the same sample after 24 h (red), as well as polyU RNA alone (1 mg ml^{-1}; green). (bottom) 2D ^{1}H-^{1}H TOCSY NMR spectra of K3 with polyU RNA forming liquid droplets (1 mM K3 + 1 mg ml^{-1} polyU RNA; black) and the same sample after 24 h (red). NMR signals, which are characteristic for K3/RNA interaction, are marked with blue asterisks.

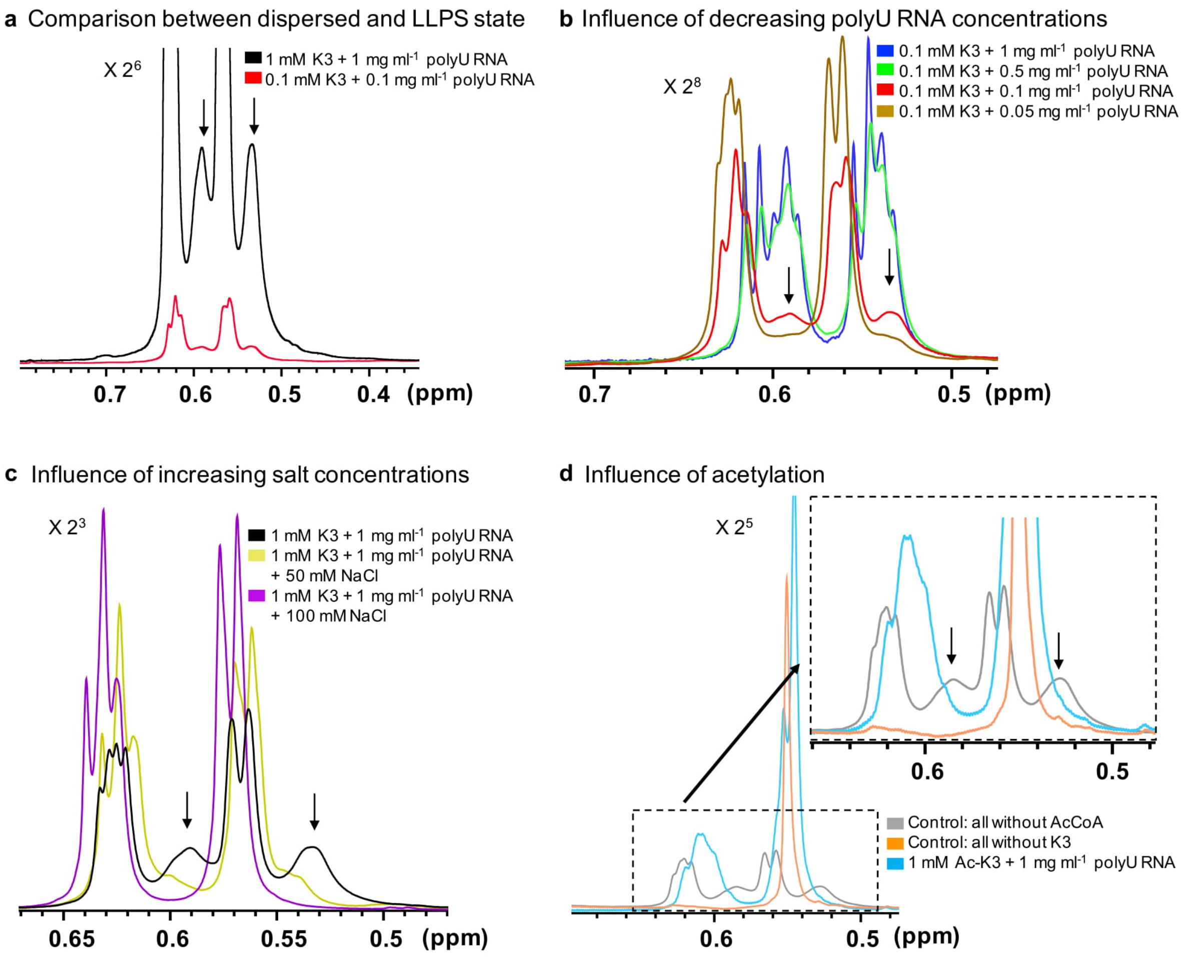

Supplementary Figure 4. Influence of peptide and RNA concentration, as well as ionic strength and acetylation, on the ^{1}H methyl resonances of the K3 peptide. **a** Comparison between the dispersed and phase-separated state at equal molar K3/RNA ratios but different total concentrations. The ^{1}H NMR resonances at ~0.53 and ~0.59 ppm remain observable at low K3/RNA concentrations, where no droplets are observed (Fig. 2c and Supplementary Fig. 2b), indicating that they are characteristic for K3/RNA interaction. **b** NMR spectra of K3 in the dispersed phase (i.e. at low peptide concentration; 0.1 mM K3) in the presence of decreasing concentration of polyU RNA. At 0.5 mg ml^{-1} and 1.0 mg ml^{-1} polyU RNA, the NMR resonances of K3 are very similar, suggesting that the interaction is largely saturated. **c** Influence of ionic strength on the ^{1}H methyl resonances of K3 (1 mM) in presence of 1 mg ml^{-1} polyU RNA. In presence of 100 mM NaCl, the ^{1}H NMR resonances at ~0.53 and ~0.59 ppm are no longer observable. **d** Acetylation of the lysine residues in K3 causes disappearance of the K3 resonances (blue spectrum), which are characteristic for the binding of K3 to polyU RNA (grey spectrum). In orange, the spectrum of the mixture containing AcCoA, CREB and polyU RNA (1.0 mg ml^{-1}), but no K3, is shown.

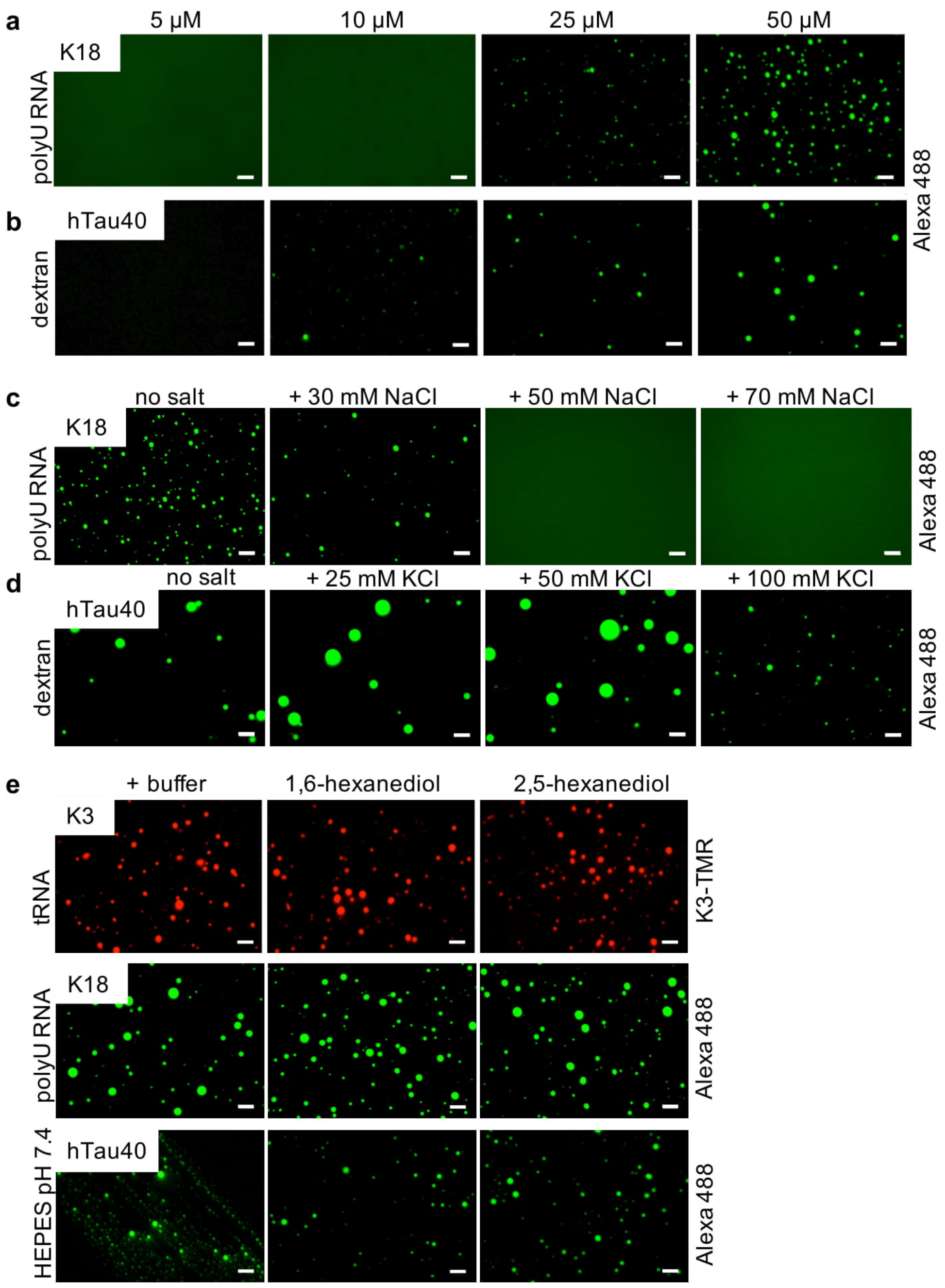

Supplementary Figure 5. Influence of protein concentration, ionic strength and aliphatic alcohols on liquid phase separation of lysine-rich polypeptides (K3, K18, hTau40). **a** Influence of protein concentration on complex coacervation of K18. PolyU RNA concentrations for 50 µM, 25 µM, 10 µM and 5 µM K18 were 125 µg ml^{-1}, 62.5 µg ml^{-1}, 25 µg ml^{-1}and 12.5 µg ml^{-1} respectively. When using 250 µg ml^{-1} polyU RNA for 50 µM K18 (or 125 µg ml^{-1} for 25 µM K18) no K18 droplets were visible. **b** Influence of protein concentration (5 µM, 10 µM, 25 µM and 50 µM) on phase separation of hTau40 at a constant percentage of dextran (10%) in 25 mM HEPES, pH 7.4. **c, d** Ionic strength dependence of liquid phase separation of (**c**) K18 (50 µM K18 + 125 µg ml^{-1} polyU RNA) and (**d**) hTau40 (50 µM hTau40 + 10 % dextran) in 25 mM HEPES, pH 7.4. **e** Phase separation in presence of 1,6- or 2,5-hexanediol (20 %): (top) K3 (1 mM K3 + 1 mg ml^{-1} tRNA in 50 mM HEPES, pH 7.4); (middle) K18 (100 µM K18 + 250 µg ml^{-1} tRNA in 25 mM HEPES, pH 7.4); (bottom) hTau40 (50 µM hTau40 in 25 mM HEPES, pH 7.4). For fluorescence microscopy, small amounts (see methods section) of TMR-K3 or Alexa 488-labeled K18 or Alexa 488-labeled hTau40 were added. Scale bars, 10 µm.

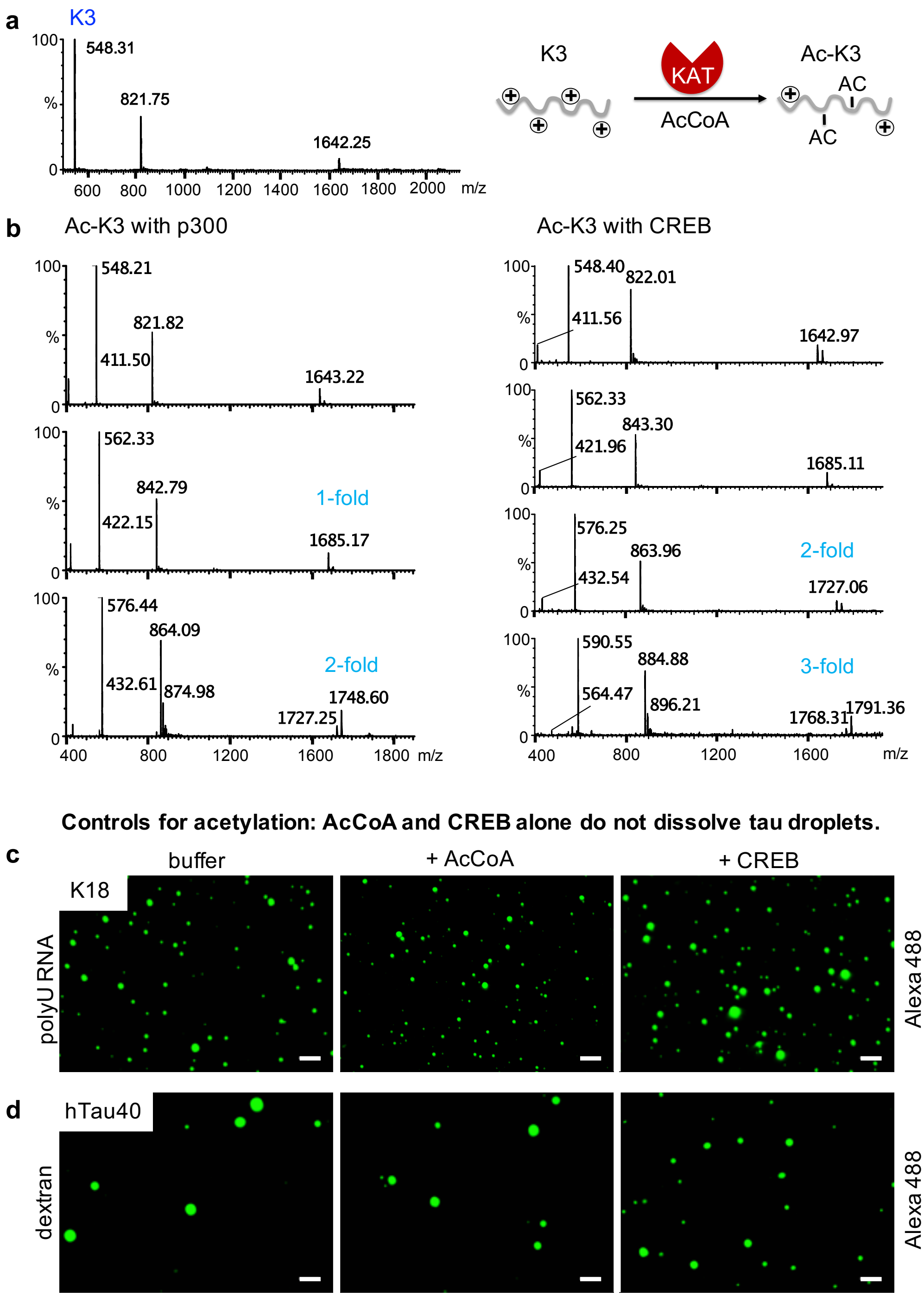

Supplementary Figure 6. Control experiments for the influence of acetylation on liquid phase separation of lysine-rich polypeptides. **a** Mass spectrum of K3 and schematic representation of the acetylation reaction. **b** Mass spectra of K3 acetylated with p300 (left) and CREB (right). Acetylation with p300 yields two acetylated groups, whereas CREB yields three acetylated sites in the K3 peptide. **c, d** Fluorescence images of (**c**) K18 (50 µM K18 + 125 µg ml^{-1} polyU RNA in 25 mM HEPES, pH 7.4) and (**d**) hTau40 (50 µM hTau40 + 10 % dextran in 25 mM HEPES, pH 7.4) control samples confirming that neither AcCoA (1 mM) nor CREB (0.62 µM) alone dissolve tau droplets either formed through coacervation with RNA (K18; **c**) or promoted by dextran (hTau40; **d**). For fluorescence microscopy, small amounts (see methods section) of Alexa 488-labeled K18 (**c**) or Alexa 488-labeled hTau40 (**d**) were added. Scale bars, 10 µm.

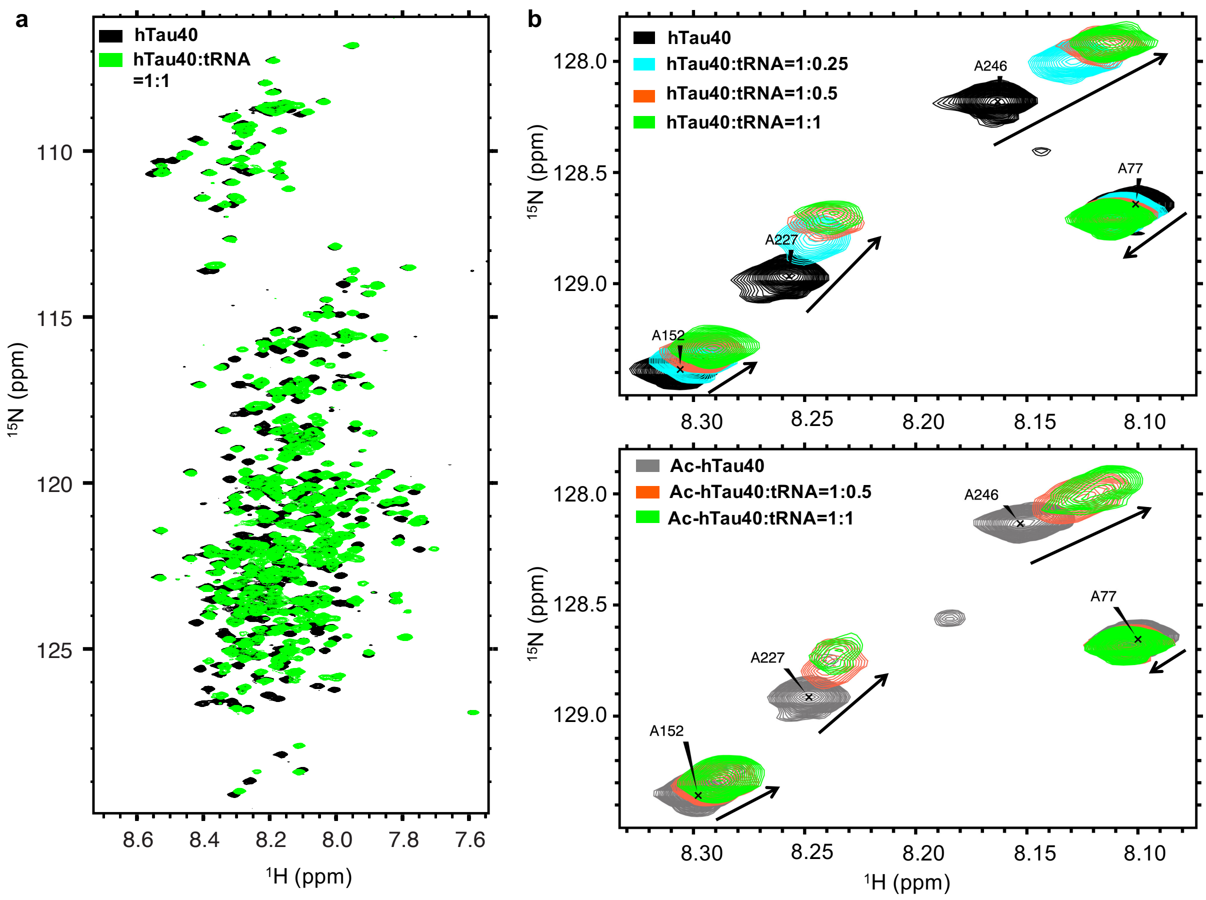

Supplementary Figure 7. Acetylation weakens binding of tau to RNA. **a** Superposition of 2D ^{1}H-^{15}N spectra of hTau40 (black) and hTau40 with RNA (molar ratio 1:1, green). **b** Superposition of selected regions of 2D ^{1}H-^{15}N spectra of hTau40 (top, black) and acetylated hTau40 (bottom, gray), respectively, for increasing molar ratios of tRNA: 0.25 (cyan), 0.5 (orange) and 1 (green).

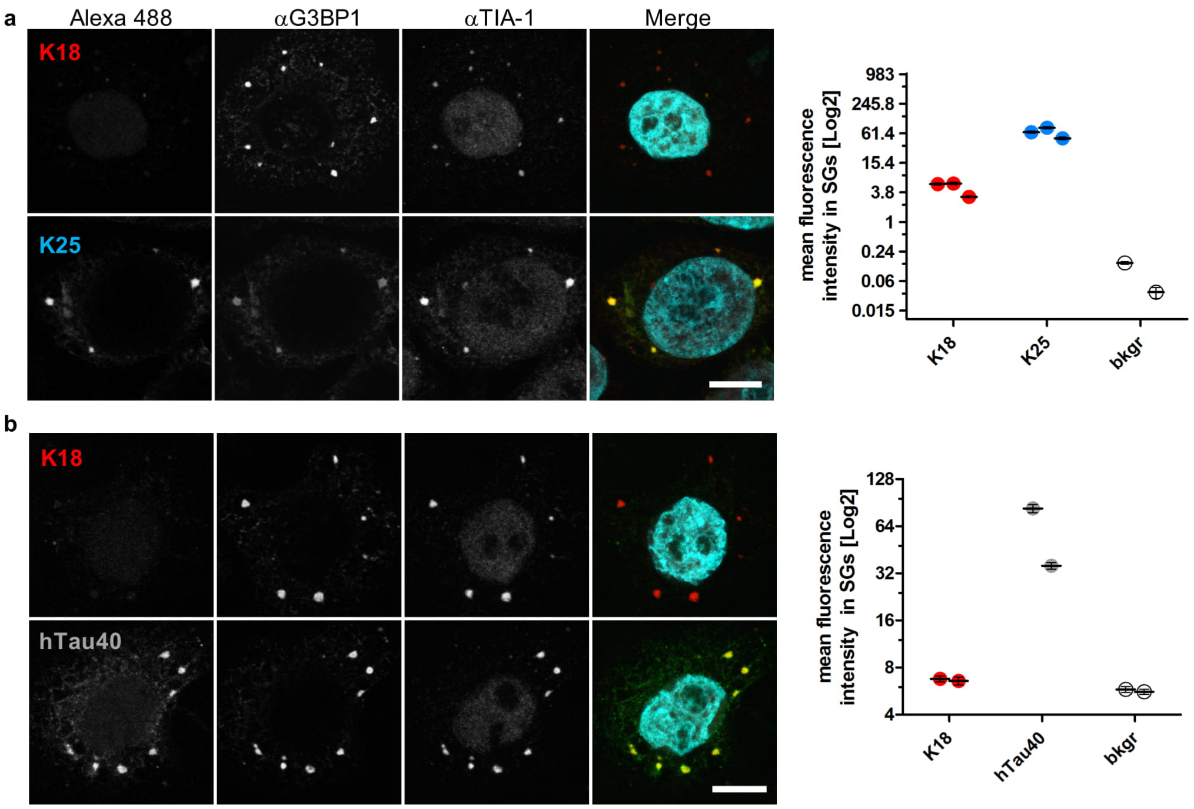

Supplementary Figure 8. Differences in SG association of tau variants with comparable DOL and cysteine-labeling. **a** SG association of K18 or K25 labeled at lysine residues with Alexa 488 (final protein concentration 400 nM; DOL 6.8 or 7, respectively) in the semi-permeabilized cell assay (see also Fig. 8b). Scale bar, 10 µm. In the merge, Alexa 488 fluorescence (green) and TIA-1 staining (red) are shown. (right panel) Quantification showing the log2-transformed mean fluorescence intensity of K18 and K25 versus background (measured in two experiments) in SGs. Three replicates (two for background control) with at least 8-10 cells and ≥45 SGs each are shown. Error bars correspond to ± SEM. **b** SG association of cysteine-labeled K18 or hTau40 (final protein concentration 400 nM; DOL 2 and 1.8 for K18 and hTau40, respectively) in the semi-permeabilized cell assay. The proteins were labeled (with Alexa Fluor 488 C5 maleimide dye) on tau's two native cysteines (C291, C322). Scale bar, 10 µm. (right panel) Quantification showing the log2-transformed mean fluorescence intensity of K18 and hTau40 versus background in SGs. Two replicates with at least 8-10 cells and ≥40 SGs each are shown. Error bars correspond to ± SEM. Source data are provided as a Source Data file.

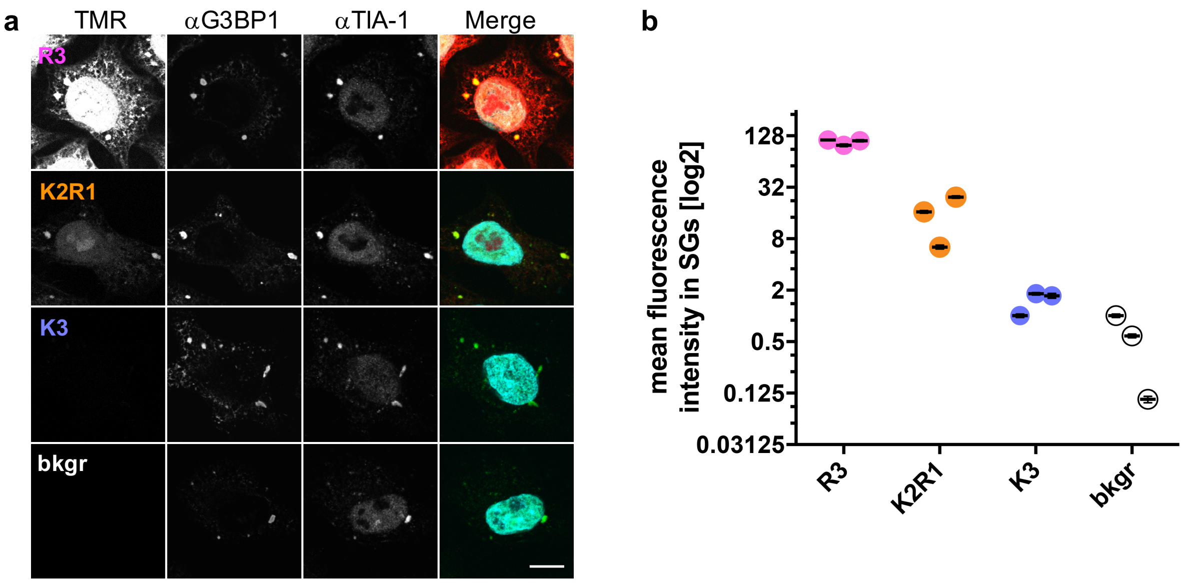

Supplementary Figure 9. Differences in SG association of TMR-labeled arginine- and lysine-rich peptides. **a** SG association of R3, K2R1 and K3 in the semi-permeabilized cell assay. Scale bar, 10 µm. In the merge, TMR-fluorescence from the peptides (red) and TIA-1 staining (AF647, displayed in green) are shown. Note, that linear enhancement for brightness and contrast in the TMR-channel was set to enhance visibility of the K2R1 peptide versus the K3 peptide, resulting in over-contrasting of the R3 peptide. **b** Quantification showing the log2-transformed mean fluorescence intensity of R3, K2R1 and K3 in SGs versus background. The mean of three independent replicates with at least 8-10 cells and ≥36 SGs each are shown. Error bars correspond to ± SEM. Source data are provided as a Source Data file.
