# Supplementary material for: Lysine/RNA-interactions drive and regulate biomolecular condensation
Source: Nat Commun. 2019 Jul 2;10:2909. doi: 10.1038/s41467-019-10792-y (PMC6606616; doi:10.1038/s41467-019-10792-y)
Supplement: Supplementary file 3 — Description of Additional Supplementary Files [file 41467_2019_10792_MOESM3_ESM.pdf]

Description of Additional Supplementary Files

File Name: Supplementary Data 1
Description: The frequency of each amino acid was measured from sequences predicted to be disordered by IUpred, given that at least 30 residues were predicted to be disordered. For each protein in the human proteome, the frequency of Lys, Arg, Tyr and Pro are listed. In each case, the set of proteins was sorted in decreasing order by amino acid frequency.

File Name: Supplementary Data 2
Description: The frequency of each amino acid was measured from sequences predicted to be disordered by IUpred, given that at least 30 residues were predicted to be disordered. For each protein in the processing body proteome, the frequency of Lys, Arg, Tyr and Pro are listed. In each case, the set of proteins was sorted in decreasing order by amino acid frequency.

File Name: Supplementary Data 3
Description: The frequency of each amino acid was measured from sequences predicted to be disordered by IUpred, given that at least 30 residues were predicted to be disordered. For each protein in the stress granule proteome, the frequency of Lys, Arg, Tyr and Pro are listed. In each case, the set of proteins was sorted in decreasing order by amino acid frequency.

File Name: Supplementary Movie 1
Description: Movie of K3/RNA droplet fusion acquired using a Leica DM6000B microscope with a 63x objective (water immersion).

File Name: Supplementary Movie 2
Description: Movie of K18/RNA droplet fusion acquired using a Leica DM6000B microscope with a 63x objective (water immersion).

File Name: Supplementary Movie 3
Description: Movie of hTau40/dextran droplet fusion acquired using a Leica DM6000B microscope with a 63x objective (water immersion).
